# Crystal structure and Hirshfeld surface analysis of (1*H*-imidazole-κ*N* ^3^)[*N*-(2-oxido­benzyl­idene)tyrosinato-κ^3^ *O*,*N*,*O*′]copper(II)

**DOI:** 10.1107/S2056989023004735

**Published:** 2023-06-02

**Authors:** Soma Suzuki, Yukihito Akiyama, Daisuke Nakane, Takashiro Akitsu

**Affiliations:** a Department of Chemistry, Faculty of Science, Tokyo University of Science, 1-3 Kagurazaka, Shinjuku-ku, Tokyo 162-8601, Japan; Universidade de Sâo Paulo, Brazil

**Keywords:** Schiff base complex, copper, amino acid, Hirshfeld analysis, crystal structure

## Abstract

The title copper(II) complex, [Cu(C_16_H_13_NO_4_)(C_3_H_4_N_2_)], consists of a tridentate ligand synthesized from l-tyrosine and salicyl­aldehyde. One imidazole mol­ecule is additionally coordinating to the copper(II) ion. The crystal structure features N—H⋯O, O—H⋯O and C—H⋯O hydrogen bonds. The Hirshfeld surface analysis indicates that the most important contributions to the packing are from H⋯H (37.9%), C⋯H (28.2%) and O⋯H/H⋯O (21.2%) contacts.

## Abstract 2

The amino acid Schiff base copper(II) title complex consists of a tridentate ligand synthesized from l-tyrosine and salicyl­aldehyde. One imidazole mol­ecule is additionally coordinating to the copper(II) ion. The crystal structure features N—H⋯O, O—H⋯O and C—H⋯O hydrogen bonds.

## 1. Chemical context

Amino acid Schiff bases, which can be easily synthesized by mixing primary amines and carbonyl components, are organic ligands having an azomethine (C=N) group. They play an important and diverse role in coordination chemistry (Qiu *et al.*, 2008 ▸; Li *et al.*, 2010 ▸; Xue *et al.*, 2009 ▸; Katsuumi *et al.*, 2020 ▸; Akiyama *et al.*, 2023 ▸). On the other hand, copper has various oxidation states, of which the divalent oxidation state is the most stable. Copper(II) ions readily form complexes and produce abundant coordination chemistry, while amino acid Schiff base–copper(II) complexes have been studied in terms of photoreaction with titanium dioxide (Takeshita *et al.*, 2015 ▸), photocatalytic reduction of hexa­valent chromium (Nakagame *et al.*, 2019 ▸), and anti­bacterial activity (Otani *et al.*, 2022 ▸). The ligand forms a tridentate chelate, but the introduction of a hydroxyl group is effective in increasing solubility in aqueous solvents (Miyagawa *et al.*, 2020 ▸). On the other hand, many similar metal complexes with an amino acid having a hydroxyl group, l-tyrosine, have been reported (Pu *et al.*, 2011 ▸; Wang *et al.*, 2005 ▸; Tan *et al.*, 2008 ▸). 
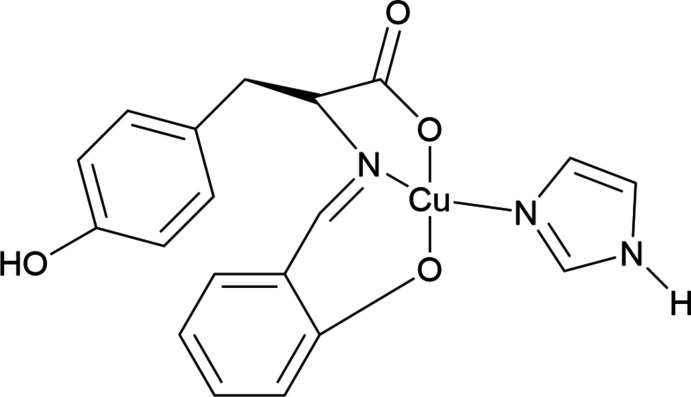


In this report we describe the crystal structure and inter­molecular inter­actions of the copper(II) complex coordinated by a tyrosine derivative and imidazole, which serves as a model for histidine residues in proteins and is effective for photoreactions with titanium dioxide. To obtain the product in higher yield than from conventional synthesis, microwave radiation was employed, although conventional synthesis may also give the same product.

## 2. Structural commentary

**Figure 1:**
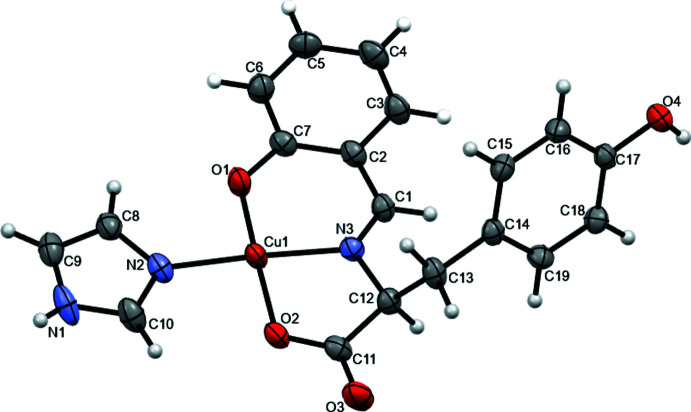
The mol­ecular structure of the title compound with ellipsoids drawn at the 50& probability level.

The mol­ecular structure of the title compound consists of a tridentate ligand occupying the equatorial plane synthesized from l-tyrosine, salicyl­aldehyde and one imidazole mol­ecule coordinating to the copper(II) center (Fig. 1 ▸). The C10—N2 distance is 1.322 (4) Å, close to a typical C=N double-bond length for an imine (Katsuumi *et al.*, 2020 ▸). The Cu1—O1 and Cu1—O2 bond lengths are 1.892 (2) and 1.947 (2) Å, respectively, close to a typical Cu—O single-bond length (Katsuumi *et al.*, 2020 ▸). The Cu1—N2 and Cu1—N3 bonds of 1.958 (2) and 1.932 (2) Å corresponds to the typical Cu—N single-bond length (Katsuumi *et al.*, 2020 ▸). These four atoms coordinating to Cu1 have similar bond distances.

## 3. Supra­molecular features

**Figure 2:**
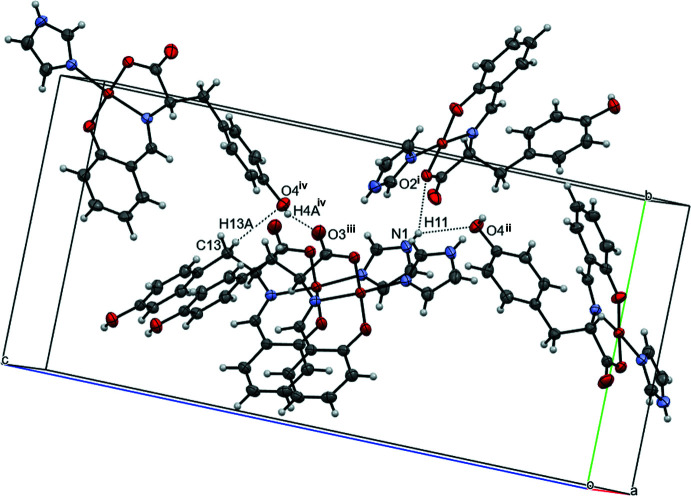
A view of the O—H⋯O, N—H⋯O and C—H⋯O hydrogen bonds, shown as dashed lines. [Symmetry codes: (i) *x* + 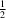 , −*y* + 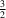 , −*z* + 1; (ii) −*x* + 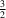 , −*y* + 1, *z* − 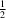 ; (iii) −*x*, *y* − 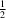 , −*z* + 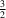 ; (iv) −*x* + 1, *y* + 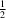 , −*z* + 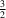 .]

Four inter­molecular O—H⋯O, N—H⋯O, C—H⋯O hydrogen bonds (Table 1 ▸ and Fig. 2 ▸) are observed in the crystal; one hydrogen bond (O2—H11⋯N1^i^; symmetry code given in Table 1 ▸) forms a chain structure along the *b*-axis direction. The other hydrogen bonds (O4—H11⋯N1^ii^, O3—H4*A*⋯O4^iii^ and O4—H13*A*⋯C13^iv^) link the mol­ecules (Fig. 2 ▸).

**Figure 3:**
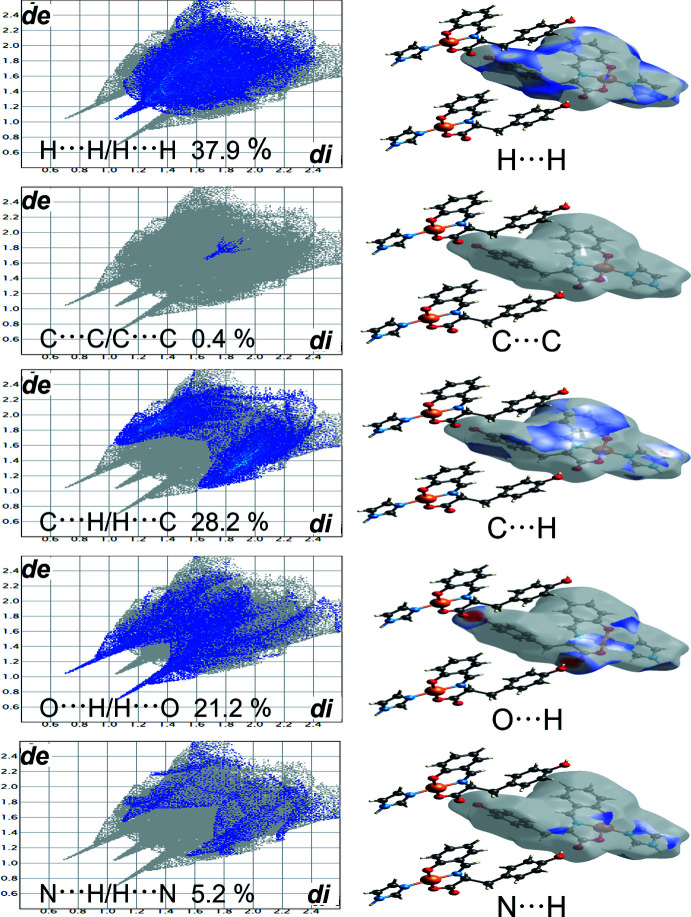
Hirshfeld surfaces mapped over *d*_norm_ and the two-dimensional fingerprint plots.

Hirshfeld surface analysis (Spackman & Jayatilaka, 2009 ▸; McKinnon *et al.*, 2007 ▸) was performed to better understand the inter­molecular inter­actions and contacts. The inter­molecular O—H⋯O hydrogen bonds are indicated by bright-red spots appearing near O3 and O4 on the Hirshfeld surfaces mapped over *d*_norm_ and by two sharp spikes of almost the same length in the region 1.6 Å < (*d*_e_ + *d*_i_) < 2.0 Å in the 2D finger plots (Fig. 3 ▸). The contributions to the packing from H⋯H, C⋯C, C⋯H/H⋯C, O⋯H/H⋯O and N⋯H/H⋯N contacts are 37.9, 0.4, 28.2, 21.2, and 5.2%, respectively. This structure is characterized by high proportions of H⋯H and C⋯H/H⋯C inter­actions, where H⋯H are van der Waals inter­actions. The force effect, C⋯H/H⋯C, is thought to arise from C—H⋯π inter­actions due to the presence of aromatic rings in the structure. The low value of C⋯C/C⋯C is the result of the low contribution of π–π stacking due to non-overlapping aromatic rings in the structure.

## 4. Database survey

A search in the Cambridge Structural Database (CSD, Version 5.41, update of March 2022; Groom *et al.*, 2016 ▸) for similar structures returned three relevant entries: {2-(4-hy­droxy­phen­yl)-2-[(3-meth­oxy-2-oxido­benzyl­idene)amino-*κ* ^2^O^2^,*N*]- propano­ato-*κO*}(1,10-phenanthroline-*κ* ^2^ *N*,*N*)copper(II) dihydrate (UNOSIA; Pu *et al.*, 2011 ▸), 2,2-bi­pyridine *N*-salicylidenetyrosinatocopper(II) (QAJTAX01; Wang *et al.*, 2005 ▸) and [(2*S*)-2-(3,5-di­chloro-2-oxidobenzyl-idene­amino)-3-(4-hy­drox­y­phen­yl)-propionato-κ^3^ *O*,*N*,*O*](di­methyl­formamide-*κO*)copper(II) (YIXKUM; Tan *et al.*, 2008 ▸).

## 5. Synthesis and crystallization

l-tyrosine (181.3 mg, 1.00 mmol) reacted with salicyl­aldehyde (125.5 mg, 1.03 mmol) in methanol (20 mL), which was treated with microwave irradiation at 358 K for 5 min to yield a yellow ligand solution. Copper(II) acetate monohydrate (200.9 mg, 1.01 mmol) was added to the ligand solution and treated with microwave irradiation at 358 K for 5 min to yield a green solution. To this green solution, imidazole (70.0 mg, 1.02 mmol) was added and treated with microwave irradiation at 358 K for 5 min to yield a dark-green solution.

For recrystallization, the solution was placed in the air at room temperature for several days, and the title complex was obtained (80.9 mg 0.195 mmol, yield 19.5%) as black needle-shaped crystals suitable for single-crystal X-ray diffraction experiments.

Elementary analysis: found: C, 54.48; H, 4.15; N, 10.11%. Calculated: C_19_H_18_CuN_3_O_4_, C, 55.00; H, 4.13; N, 10.13%. IR (KBr): 1059 cm^−1^ (*m*), 1085 cm^−1^ (*w*), 1128 cm^−1^ (*w*), 1149 cm^−1^ (*m*), 1225 cm^−1^ (*w*), 1271 cm^−1^ (*m*), 1370 cm^−1^ (*w*), 1372 cm^−1^ (*w*), 1378 cm^−1^ (*m*), 1384 cm^−1^ (*w*), 1448 cm^−1^ (*s*, C=C double bond), 1516 cm^−1^ (*m*), 1610 cm^−1^ (*s*, C=O double bond), 1625 cm^−1^(*s*, C=N double bond), 3159 cm^−1^ (*br*), 3214 cm^−1^ (*br*), 3297 cm^−1^ (*br*, O⋯H). UV–vis (MeOH): 269 nm (ɛ= 13636 L mol^−1^ cm^−1^, π–π^*^); 368 nm (ɛ = 5636 L mol^−1^ cm^−1^, *n*–π^*^); 618 nm (ɛ = 135 L mol^−1^ cm^−1^, *d*–*d*).

## 6. Refinement

Crystal data, data collection and structure refinement details are summarized in Table 2 ▸. All C-bound H atoms were placed on geometrically calculated positions (C—H = 0.93–0.98 Å) and were constrained using a riding model with *U*_iso_(H) = 1.2*U*_eq_(C) for *R*_2_CH and *R*_3_CH H atoms and 1.5*U*_eq_(C) for the methyl H atoms. The O-bound H14 atom was located based on a difference-Fourier map and refined freely as an isotropic atom. The N-bound H atoms were located in a difference-Fourier map. Atom H11 of the imidazole ring was refined freely as an isotropic atom.

## Supplementary Material

Crystal structure: contains datablock(s) global, I. DOI: 10.1107/S2056989023004735/ex2071sup1.cif

Structure factors: contains datablock(s) I. DOI: 10.1107/S2056989023004735/ex2071Isup2.hkl

Supporting information file. DOI: 10.1107/S2056989023004735/ex2071sup3.tif

CCDC reference: 2266335

Additional supporting information:  crystallographic information; 3D view; checkCIF report

## Figures and Tables

**Table 1:** Hydrogen-bond geometry (Å, °)

*D*—H⋯*A*	*D*—H	H⋯*A*	*D*⋯*A*	*D*—H⋯*A*
N1—H11⋯O2^i^	0.75 (4)	2.40 (4)	3.050 (3)	147 (3)
N1—H11⋯O4^ii^	0.75 (4)	2.48 (3)	3.058 (3)	136 (3)
O4—H4*A*⋯O3^iii^	0.74	1.98	2.666 (3)	154
C13—H13*A*⋯O4^iv^	0.99	2.58	3.364 (3)	136

Symmetry codes: (i) 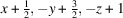 ; (ii) 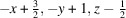 ; (iii) 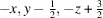 ; (iv) 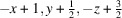 .

**Table 2:** Experimental details

Crystal data	
Chemical formula	[Cu(C_16_H_13_NO_4_)(C_3_H_4_N_2_)]
*M*_r_	414.89
Crystal system, space group	Orthorhombic, *P*2_1_2_1_2_1_
Temperature (K)	173
*a*, *b*, *c* (Å)	5.5005 (2), 12.1363 (5), 26.147 (1)
*V* (Å^3^)	1745.46 (12)
*Z*	4
Radiation type	Mo *K*α
μ (mm^−1^)	1.28
Crystal size (mm)	0.50 × 0.30 × 0.20

Data collection	
Diffractometer	Bruker D8 QUEST
Absorption correction	Multi-scan (*SADABS*; Krause *et al.*, 2015 ▸)
*T*_min_, *T*_max_	0.55, 0.78
No. of measured, independent and observed [*I* > 2σ(*I*)] reflections	19853, 3571, 3501
*R*_int_	0.047
(sin θ/λ)_max_ (Å^−1^)	0.626

Refinement	
*R*[*F* ^2^ > 2σ(*F* ^2^)], *wR*(*F* ^2^), *S*	0.025, 0.062, 1.06
No. of reflections	3571
No. of parameters	251
H-atom treatment	H atoms treated by a mixture of independent and constrained refinement
Δρ_max_, Δρ_min_ (e Å^−3^)	0.33, −0.21
Absolute structure	Refined as an inversion twin
Absolute structure parameter	0.017 (12)

Computer programs: *APEX2* and *SAINT* V8.40B (Bruker, 2019 ▸), *SHELXT2018/2* (Sheldrick, 2015*a* ▸), *SHELXL2018/3* (Sheldrick, 2015*b* ▸) and *ShelXle* (Hübschle *et al.*, 2011 ▸).

## supplementary crystallographic information

### Crystal data

**Table:**

[Cu(C_16_H_13_NO_4_)(C_3_H_4_N_2_)]	*D*_x_ = 1.579 Mg m^−^^3^
*M**_r_* = 414.89	Mo *K*α radiation, λ = 0.71073 Å
Orthorhombic, *P*2_1_2_1_2_1_	Cell parameters from 9965 reflections
*a* = 5.5005 (2) Å	θ = 2.9–26.4°
*b* = 12.1363 (5) Å	µ = 1.28 mm^−^^1^
*c* = 26.147 (1) Å	*T* = 173 K
*V* = 1745.46 (12) Å^3^	Prism, black
*Z* = 4	0.50 × 0.30 × 0.20 mm
*F*(000) = 852	

### Data collection

**Table:**

Bruker D8 QUEST diffractometer	3501 reflections with *I* > 2σ(*I*)
Detector resolution: 7.3910 pixels mm^-1^	*R*_int_ = 0.047
Absorption correction: multi-scan (SADABS; Krause *et al.*, 2015)	θ_max_ = 26.4°, θ_min_ = 1.9°
*T*_min_ = 0.55, *T*_max_ = 0.78	*h* = −6→6
19853 measured reflections	*k* = −15→15
3571 independent reflections	*l* = −32→32

### Refinement

**Table:**

Refinement on *F*^2^	Hydrogen site location: mixed
Least-squares matrix: full	H atoms treated by a mixture of independent and constrained refinement
*R*[*F*^2^ > 2σ(*F*^2^)] = 0.025	*w* = 1/[σ^2^(*F*_o_^2^) + (0.0276*P*)^2^ + 0.2834*P*] where *P* = (*F*_o_^2^ + 2*F*_c_^2^)/3
*wR*(*F*^2^) = 0.062	(Δ/σ)_max_ = 0.001
*S* = 1.06	Δρ_max_ = 0.33 e Å^−^^3^
3571 reflections	Δρ_min_ = −0.21 e Å^−^^3^
251 parameters	Absolute structure: Refined as an inversion twin
0 restraints	Absolute structure parameter: 0.017 (12)

### Special details

**Table:**

Geometry. All esds (except the esd in the dihedral angle between two l.s. planes) are estimated using the full covariance matrix. The cell esds are taken into account individually in the estimation of esds in distances, angles and torsion angles; correlations between esds in cell parameters are only used when they are defined by crystal symmetry. An approximate (isotropic) treatment of cell esds is used for estimating esds involving l.s. planes.
Refinement. Refined as a 2-component inversion twin.

### Fractional atomic coordinates and isotropic or equivalent isotropic displacement parameters (Å^2^)

**Table:**

	*x*	*y*	*z*	*U*_iso_*/*U*_eq_	
Cu1	0.67468 (5)	0.46981 (2)	0.56047 (2)	0.02401 (11)	
O1	0.8939 (3)	0.35005 (15)	0.55615 (7)	0.0334 (4)	
N1	0.9329 (6)	0.6643 (2)	0.44552 (9)	0.0420 (6)	
H11	0.951 (6)	0.717 (3)	0.4317 (12)	0.031 (8)*	
C1	0.5421 (5)	0.3165 (2)	0.63934 (9)	0.0263 (5)	
H1	0.429138	0.291841	0.664328	0.032*	
O2	0.4294 (3)	0.58526 (14)	0.56409 (7)	0.0287 (4)	
N2	0.8346 (4)	0.54082 (17)	0.50227 (7)	0.0288 (4)	
C2	0.7397 (5)	0.2446 (2)	0.62716 (9)	0.0266 (5)	
N3	0.5042 (4)	0.41212 (17)	0.61927 (8)	0.0247 (4)	
O3	0.1144 (4)	0.64649 (19)	0.60736 (8)	0.0437 (5)	
C3	0.7677 (6)	0.1496 (2)	0.65788 (10)	0.0327 (6)	
H3	0.654313	0.13602	0.684559	0.039*	
O4	0.3089 (4)	0.22066 (16)	0.85680 (7)	0.0341 (4)	
H4A	0.191 (7)	0.191 (3)	0.8583 (9)	0.051*	
C4	0.9544 (6)	0.0768 (2)	0.65012 (11)	0.0355 (6)	
H4	0.973698	0.014587	0.671828	0.043*	
C5	1.1151 (5)	0.0952 (2)	0.60999 (11)	0.0350 (6)	
H5	1.244308	0.044786	0.604282	0.042*	
C6	1.0904 (5)	0.1854 (2)	0.57830 (11)	0.0337 (6)	
H6	1.200419	0.194846	0.550635	0.04*	
C7	0.9050 (5)	0.2637 (2)	0.58620 (9)	0.0271 (5)	
C8	1.0473 (5)	0.5091 (2)	0.47797 (9)	0.0317 (6)	
H8	1.136848	0.443916	0.484952	0.038*	
C9	1.1065 (5)	0.5856 (2)	0.44292 (10)	0.0373 (6)	
H9	1.243185	0.584623	0.420719	0.045*	
C10	0.7719 (6)	0.6358 (2)	0.48134 (10)	0.0372 (7)	
H10	0.632492	0.677619	0.490455	0.045*	
C11	0.2751 (5)	0.5780 (2)	0.60006 (9)	0.0280 (5)	
C12	0.2973 (5)	0.4790 (2)	0.63643 (8)	0.0262 (5)	
H12	0.145376	0.433906	0.634481	0.031*	
C13	0.3284 (5)	0.5233 (2)	0.69154 (8)	0.0301 (5)	
H13A	0.485063	0.563191	0.693498	0.036*	
H13B	0.197503	0.577417	0.698212	0.036*	
C14	0.3233 (5)	0.43791 (19)	0.73347 (8)	0.0248 (5)	
C15	0.5135 (5)	0.4309 (2)	0.76836 (10)	0.0290 (6)	
H15	0.651326	0.477154	0.764186	0.035*	
C16	0.5065 (5)	0.3581 (2)	0.80895 (10)	0.0305 (5)	
H16	0.638341	0.354933	0.832366	0.037*	
C17	0.3077 (5)	0.28973 (19)	0.81552 (8)	0.0251 (5)	
C18	0.1184 (5)	0.2928 (2)	0.78049 (9)	0.0276 (5)	
H18	−0.016298	0.244436	0.784071	0.033*	
C19	0.1271 (5)	0.3673 (2)	0.74005 (9)	0.0275 (5)	
H19	−0.004041	0.36992	0.716438	0.033*	

### Atomic displacement parameters (Å^2^)

**Table:**

	*U*^11^	*U*^22^	*U*^33^	*U*^12^	*U*^13^	*U*^23^
Cu1	0.03258 (17)	0.02037 (15)	0.01908 (14)	0.00257 (12)	0.00109 (11)	0.00198 (11)
O1	0.0418 (10)	0.0297 (9)	0.0287 (9)	0.0086 (8)	0.0108 (8)	0.0089 (8)
N1	0.0750 (19)	0.0271 (12)	0.0239 (11)	−0.0099 (12)	0.0061 (12)	0.0052 (10)
C1	0.0339 (14)	0.0223 (12)	0.0227 (11)	0.0004 (11)	0.0033 (10)	−0.0008 (9)
O2	0.0396 (9)	0.0256 (8)	0.0207 (8)	0.0061 (7)	−0.0004 (8)	0.0028 (7)
N2	0.0411 (12)	0.0236 (10)	0.0217 (9)	−0.0016 (11)	−0.0006 (9)	0.0013 (8)
C2	0.0361 (14)	0.0201 (11)	0.0236 (11)	0.0016 (10)	−0.0001 (9)	−0.0002 (9)
N3	0.0295 (11)	0.0218 (10)	0.0228 (9)	0.0034 (9)	0.0013 (8)	0.0001 (8)
O3	0.0537 (14)	0.0427 (11)	0.0346 (10)	0.0245 (11)	0.0055 (9)	0.0059 (9)
C3	0.0434 (16)	0.0243 (12)	0.0304 (13)	0.0023 (12)	0.0035 (11)	0.0046 (10)
O4	0.0361 (10)	0.0364 (10)	0.0297 (9)	0.0009 (9)	0.0002 (9)	0.0098 (8)
C4	0.0489 (17)	0.0218 (12)	0.0359 (14)	0.0042 (12)	−0.0023 (13)	0.0059 (11)
C5	0.0378 (15)	0.0242 (13)	0.0429 (15)	0.0090 (12)	−0.0013 (12)	−0.0003 (11)
C6	0.0377 (15)	0.0296 (14)	0.0340 (13)	0.0036 (12)	0.0068 (11)	0.0019 (11)
C7	0.0338 (13)	0.0208 (12)	0.0268 (12)	0.0019 (10)	−0.0011 (10)	−0.0007 (10)
C8	0.0332 (14)	0.0357 (14)	0.0261 (12)	−0.0030 (11)	−0.0010 (10)	0.0016 (10)
C9	0.0435 (15)	0.0423 (15)	0.0261 (12)	−0.0105 (13)	0.0031 (12)	−0.0006 (12)
C10	0.0593 (19)	0.0252 (12)	0.0272 (12)	0.0028 (13)	0.0049 (12)	0.0005 (10)
C11	0.0359 (14)	0.0252 (12)	0.0229 (11)	0.0050 (11)	−0.0043 (10)	−0.0033 (9)
C12	0.0316 (12)	0.0231 (11)	0.0239 (11)	0.0035 (12)	0.0029 (9)	−0.0017 (9)
C13	0.0444 (14)	0.0223 (11)	0.0236 (11)	0.0016 (13)	0.0054 (11)	−0.0008 (10)
C14	0.0296 (13)	0.0230 (11)	0.0216 (10)	0.0025 (10)	0.0044 (10)	−0.0027 (8)
C15	0.0257 (13)	0.0326 (13)	0.0288 (12)	−0.0034 (11)	0.0045 (10)	−0.0022 (10)
C16	0.0259 (12)	0.0395 (14)	0.0261 (12)	0.0004 (12)	−0.0008 (10)	0.0011 (11)
C17	0.0297 (12)	0.0236 (11)	0.0220 (10)	0.0035 (11)	0.0027 (10)	0.0002 (9)
C18	0.0279 (13)	0.0255 (12)	0.0296 (12)	−0.0043 (10)	0.0016 (10)	0.0001 (10)
C19	0.0279 (14)	0.0307 (13)	0.0239 (11)	−0.0010 (11)	−0.0035 (9)	−0.0007 (10)

### Geometric parameters (Å, º)

**Table:**

Cu1—O1	1.8919 (18)	C3—C4	1.371 (4)
Cu1—N3	1.932 (2)	O4—C17	1.367 (3)
Cu1—O2	1.9473 (17)	C4—C5	1.390 (4)
Cu1—N2	1.958 (2)	C5—C6	1.379 (4)
O1—C7	1.311 (3)	C6—C7	1.410 (4)
N1—C10	1.335 (4)	C8—C9	1.345 (4)
N1—C9	1.352 (4)	C11—C12	1.537 (3)
C1—N3	1.290 (3)	C12—C13	1.547 (3)
C1—C2	1.430 (4)	C13—C14	1.509 (3)
O2—C11	1.270 (3)	C14—C19	1.389 (4)
N2—C10	1.322 (4)	C14—C15	1.390 (4)
N2—C8	1.386 (4)	C15—C16	1.381 (4)
C2—C3	1.413 (3)	C16—C17	1.383 (4)
C2—C7	1.424 (4)	C17—C18	1.388 (4)
N3—C12	1.468 (3)	C18—C19	1.392 (4)
O3—C11	1.228 (3)		
			
O1—Cu1—N3	94.49 (8)	O1—C7—C6	119.0 (2)
O1—Cu1—O2	175.72 (8)	O1—C7—C2	123.5 (2)
N3—Cu1—O2	83.45 (8)	C6—C7—C2	117.5 (2)
O1—Cu1—N2	90.31 (8)	C9—C8—N2	109.0 (3)
N3—Cu1—N2	174.98 (9)	C8—C9—N1	106.4 (3)
O2—Cu1—N2	91.86 (8)	N2—C10—N1	110.1 (3)
C7—O1—Cu1	127.44 (16)	O3—C11—O2	123.3 (2)
C10—N1—C9	108.7 (2)	O3—C11—C12	119.3 (2)
N3—C1—C2	125.5 (2)	O2—C11—C12	117.3 (2)
C11—O2—Cu1	116.69 (16)	N3—C12—C11	107.73 (19)
C10—N2—C8	105.8 (2)	N3—C12—C13	113.0 (2)
C10—N2—Cu1	126.0 (2)	C11—C12—C13	108.25 (19)
C8—N2—Cu1	127.85 (18)	C14—C13—C12	115.8 (2)
C3—C2—C7	119.4 (2)	C19—C14—C15	117.7 (2)
C3—C2—C1	117.0 (2)	C19—C14—C13	121.9 (2)
C7—C2—C1	123.6 (2)	C15—C14—C13	120.3 (2)
C1—N3—C12	119.8 (2)	C16—C15—C14	121.5 (3)
C1—N3—Cu1	124.84 (18)	C15—C16—C17	120.1 (3)
C12—N3—Cu1	114.78 (15)	O4—C17—C16	117.5 (2)
C4—C3—C2	121.6 (3)	O4—C17—C18	122.8 (2)
C3—C4—C5	118.9 (2)	C16—C17—C18	119.7 (2)
C6—C5—C4	121.3 (3)	C17—C18—C19	119.6 (2)
C5—C6—C7	121.2 (2)	C14—C19—C18	121.4 (2)

### Hydrogen-bond geometry (Å, º)

**Table:**

*D*—H···*A*	*D*—H	H···*A*	*D*···*A*	*D*—H···*A*
N1—H11···O2^i^	0.75 (4)	2.40 (4)	3.050 (3)	147 (3)
N1—H11···O4^ii^	0.75 (4)	2.48 (3)	3.058 (3)	136 (3)
O4—H4*A*···O3^iii^	0.74	1.98	2.666 (3)	154
C13—H13*A*···O4^iv^	0.99	2.58	3.364 (3)	136

Symmetry codes: (i) *x*+1/2, −*y*+3/2, −*z*+1; (ii) −*x*+3/2, −*y*+1, *z*−1/2; (iii) −*x*, *y*−1/2, −*z*+3/2; (iv) −*x*+1, *y*+1/2, −*z*+3/2.
