# Supplementary figures and images for: Crystal structure and Hirshfeld surface analysis of (1H-imidazole-κN 3)[N-(2-oxido­benzyl­idene)tyrosinato-κ3 O,N,O′]copper(II)
Source: Acta Crystallogr E Crystallogr Commun. 2023 Jun 2;79(Pt 7):596–9. doi: 10.1107/S2056989023004735 (PMC10439432; doi:10.1107/S2056989023004735)

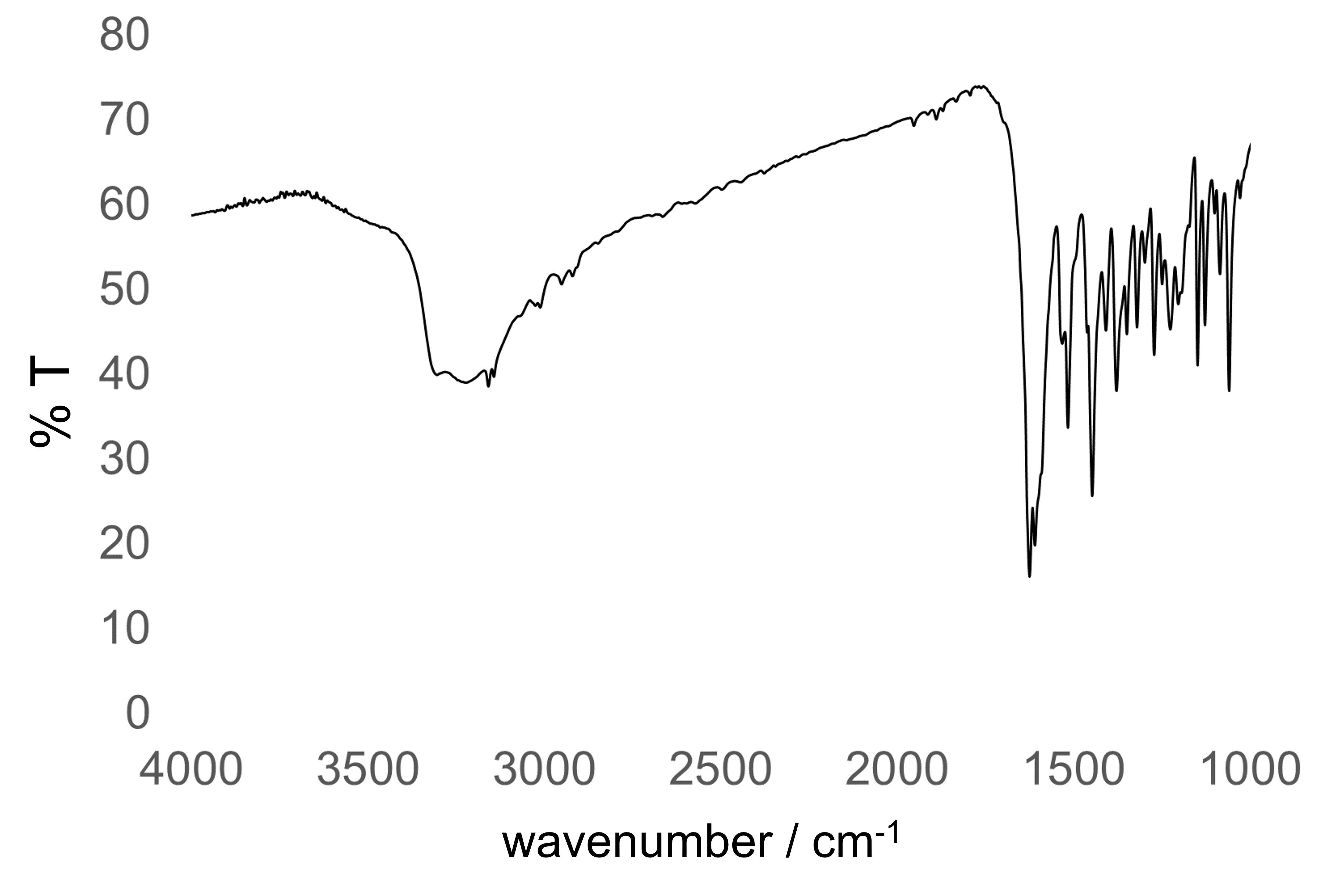

Supplement: Supplementary file 3 [file e-79-00596-sup3.tif]
